# Draft Genome Sequences of the Ferric Iron-Reducing *Geobacter* sp. Strains AOG1 and AOG2, Isolated from Enrichment Cultures on Crystalline Iron(III) Oxides

**DOI:** 10.1128/MRA.00913-21

**Published:** 2021-11-04

**Authors:** Yong Guo, Tomo Aoyagi, Tomoyuki Hori

**Affiliations:** a Environmental Management Research Institute, National Institute of Advanced Industrial Science and Technology (AIST), Tsukuba, Ibaraki, Japan; Montana State University

## Abstract

Here, we report the draft genome sequences of two *Geobacter* sp. strains, AOG1 and AOG2, isolated from enrichment cultures using crystalline Fe(III) oxides as electron acceptors. Strains AOG1 and AOG2 possess numerous genes encoding multiheme *c*-type cytochromes and *pilA-N* genes encoding the pilin monomer of nanowires in their genomes.

## ANNOUNCEMENT

*Geobacter* species are of significance in understanding direct electron transfer from microbes to inorganic acceptors located outside the cells, such as crystalline Fe(III) oxides (1, 2). Recent multidisciplinary research has reported that protein nanowires and multiheme *c*-type cytochromes are responsible for extracellular electron transport (3–7). Yet the mechanisms underlying crystalline Fe(III) oxide reduction are largely unknown. In our previous work, the ferric iron-reducing *Geobacter* sp. strains AOG1 and AOG2 were isolated from enrichment cultures, using rice paddy and wetland soils as the inocula and lepidocrocite and magnetite as the electron acceptors, respectively, indicating their involvement in the reduction of these crystalline Fe(III) oxides (8). Here, we report the draft genome sequences of the two strains, AOG1 and AOG2.

The genome sequences of strains AOG1 and AOG2 were obtained as previously described (9). Briefly, the strains were grown anaerobically on Fe(III)-nitrilotriacetic acid (NTA) as the electron acceptor, and their genomic DNA was extracted using phenol extraction with chemical cell lysis (10). For both strains, two kinds of DNA libraries were generated; one was a paired-end library (insert size, ∼500 bp), prepared using a NEBNext Ultra DNA library prep kit for Illumina (New England BioLabs, Ipswich, MA, USA), while the other was a mate-pair library (insert size, ∼4,000 bp), prepared using a Nextera mate pair sample prep kit (Illumina, San Diego, CA, USA). These libraries were sequenced on an Illumina MiSeq platform with 250-bp paired-end reads. Low-quality reads were removed using Sickle software v1.33 (https://github.com/najoshi/sickle/releases/tag/v1.33) with the default quality score threshold (Q > 20). The high-quality reads were preassembled using Unicycler v0.4.8 (11) with default parameters and then assembled using the trusted-contigs tool in SPAdes v3.13.0 software (12) with a coverage cutoff of 30-fold. GenoFinisher v2.1 software (http://www.ige.tohoku.ac.jp/joho/gf/index.php) was used to close the genome sequences; however, unclosed scaffolds of 3.68 Mb and 3.92 Mb were obtained for strains AOG1 and AOG2, respectively. A summary of the assembly statistics of the strains is shown in Table 1. The genome sequences of both strains were annotated using DFAST v1.2.2 with all the built-in databases (13). The genome completeness was assessed using the CheckM lineage workflow (14). Multiheme *c*-type cytochromes were identified using a Python script, as previously reported (15, 16).

**TABLE 1:** Assembly information, genome features, and data accession numbers of the *Geobacter* sp. strains AOG1 and AOG2

Characteristic	Data for strain:	
	AOG1	AOG2
No. of paired-end reads	1,950,584	2,122,640
No. of mate-pair reads	800,232	725,682
Assembly level	Scaffold	Scaffold
Genome size (bp)	3,677,207	3,921,358
G+C content (%)	57.4	57.2
Genome coverage (×)	157	211
No. of scaffolds	1	1
*N*_50_ (bp)	3,677,207	3,921,358
Genome completeness (%)	99.95	99.35
No. of rRNAs	6	6
No. of tRNAs	51	52
No. of CDSs	3,292	3,515
No. of genes for multiheme *c*-type cytochrome	51	49
No. of copies of *pilA-N*	1	2
DDBJ/ENA/GenBank accession no.	BLIZ01000001	BLJA01000001
SRA accession no.	DRA009323	DRA009324
SRA accession no. for paired-end reads	DRR200236	DRR200239
SRA accession no. for mate-pair reads	DRR200237	DRR200238
BioSample accession no.	SAMD00196143	SAMD00196144

The draft genome sequence of strain AOG1 contains 2 rRNA operons, 51 tRNA loci, and 3,292 protein-coding sequences (CDSs), with 99.95% genome completeness, whereas that of strain AOG2 contains 2 rRNA operons, 52 tRNA loci, and 3,515 CDSs, with 99.35% completeness (Table 1). The draft genome sequences of AOG1 and AOG2 include at least 51 and 49 genes encoding multiheme *c*-type cytochromes, as well as 1 and 2 copies of the *pilA-N* gene encoding the pilin monomer of nanowires, respectively (Table 1). The draft genome sequences of *Geobacter* sp. strains AOG1 and AOG2 will be useful for a comprehensive understanding of the microbial reduction of crystalline Fe(III) oxides.

### Data availability.

The genome sequences of *Geobacter* sp. strains AOG1 and AOG2 have been deposited at DDBJ/ENA/GenBank under the BioProject accession number PRJDB9051, with the individual accession numbers shown in Table 1. The raw data sets are available under the SRA accession numbers DRA009323 and DRA009324 for strains AOG1 and AOG2, respectively.
